# *Ascaris lumbricoides*: To Expect the Unexpected during a Routine Colonoscopy

**DOI:** 10.1155/2013/579464

**Published:** 2013-06-11

**Authors:** Kalyan Kanneganti, Jasbir S. Makker, Prospere Remy

**Affiliations:** ^1^Division of Gastroenterology, Bronx Lebanon Hospital Center, Bronx, NY 10457, USA; ^2^Department of Internal Medicine, Bronx Lebanon Hospital Center, Bronx, NY 10457, USA

## Abstract

*Ascaris lumbricoides* is a common nematode infecting humans worldwide with increased prevalence in tropical and subtropical areas of less developed countries. Recently, it has been estimated that over one billion individuals are infected with ascariasis worldwide with 7% in USA. Although most of these cases are due to increasing immigration and travel outside America it is worth recognizing that prevalence of ascariasis is high in southeastern parts of USA due to their temperate climate. Infections of *A. lumbricoides* are largely asymptomatic, and hence a large population of people carrying this worm remains undetected for years until they develop some symptoms. Due to a large group of asymptomatic individuals with intestinal ascariasis, these worms are occasionally and unexpectedly identified during routine endoscopic procedures. Here, we present a case of an intestinal ascariasis found during routine colonoscopy in an African-American man from the Bronx with perianal itching. He denied any history of travel outside USA but reported frequent visits to South Carolina. This case illustrates the fact that ascariasis should be suspected even if immigration or travel outside USA is not involved. It should be suspected even in cases of travel within USA to the south east where endemic cases are reported.

## 1. Introduction

In the USA, after hookworm and whipworm, ascariasis is the third most common helminthic infection [1]. Economic burden of ascariasis in USA accounts for about 60,000 disability-adjusted life years (DALYs) [2]. According to a WHO estimate in the year 2003, prevalence of ascariasis is 1222 millions worldwide with 84 million cases in USA [3]. Most of these infections are asymptomatic with symptoms largely restricted to individuals with high worm load [1].

## 2. Case Report

A 58-year-old man with medical history significant for diabetes mellitus and hypertension presented to our gastroenterology clinic with symptoms of perianal itching for two months. He denied any symptoms of abdominal pain, nausea, vomiting, diarrhea, or gastrointestinal bleeding. He denied having similar symptoms in the past. He also denied any family member having similar symptoms. 

Personal history was significant for six-pack year cigarette smoking history and social alcohol use. He denied any drug allergies. He had travelled to South Carolina several times. On initial examination, blood pressure was 137/65 mmHg, pulse rate 66 beats per minute, respiration rate 16 per minute, and temperature 98 degree Fahrenheit. Physical exam including the rectal exam was normal. 

**Figure 1:**
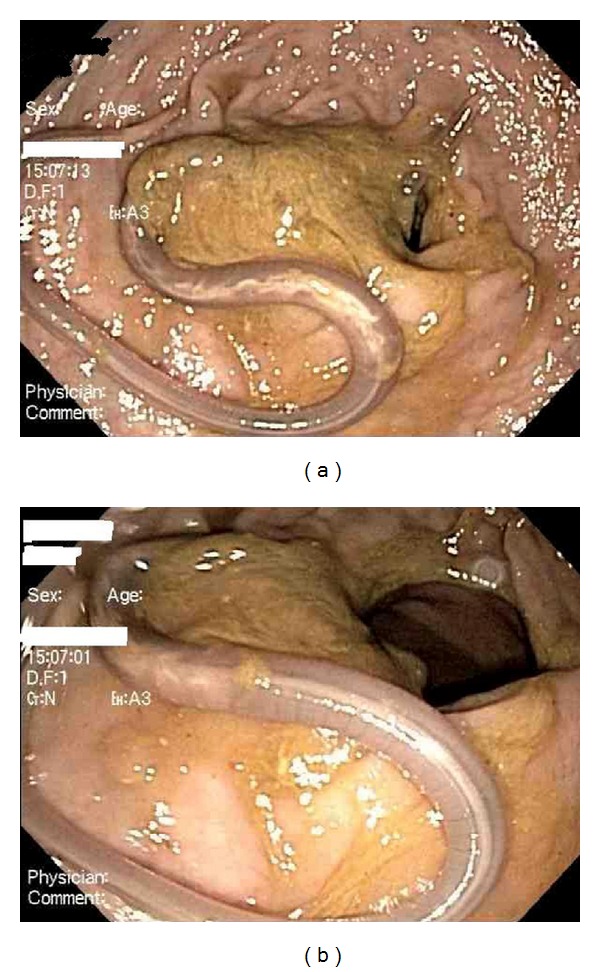
(a) *A. lumbricoides* seen during routine colonoscopy. (b) *A. lumbricoides* seen during routine colonoscopy.

During colonoscopy, one round live parasite was found in the rectum (Figures 1(a) and 1(b)). Colonoscopy also revealed multiple diminutive polyps in the rectosigmoid colon with one 2 mm polyp each in the transverse colon and the descending colon. Removal of parasite was accomplished with regular forceps. The round intestinal parasite recovered from the rectum was then identified as *A. lumbricoides*. Biopsy of all the polyps was also performed which turned out to be hyperplastic polyp for all the polyps. Anthelmintic treatment with Albendazole was started, and currently our patient lives a healthy and asymptomatic life with his spouse.

## 3. Discussion

*A. lumbricoides* (the common round worm) infection is a common parasitic infection distributed worldwide. It is estimated that about 1222 millions are infected with ascariasis worldwide [3]. Its occurrence is more common in tropical and subtropical areas of less developed countries due to poor hygiene and sanitary conditions prevalent in these countries. Prevalence of ascariasis is the highest in Western Pacific region (705 millions), followed by Southeast Asia (237 millions) and Africa (173 millions) [3]. In the USA, after hookworm and whipworm, ascariasis is the third most common helminthic infection [1] with an estimated prevalence of 84 millions [3]. Studies on Minnesota refugees [4] and Massachusetts refugees [5] revealed high prevalence of parasitic infections including ascariasis among immigrants. As a result of these studies, parasitic infections are often attributed to immigration and travel outside USA. However, southeastern parts of USA, due to their temperate climate, are endemic with high prevalence of ascariasis [6]. Hence, intestinal ascariasis should always be kept in the differential diagnosis for someone presenting with abdominal symptoms irrespective of travel history.

Humans are the definitive host for *A. lumbricoides* infection, and feco-oral transmission of embryonated eggs remains the main mode of transmission. A female *A. lumbricoides* living in the small intestine lays about 200,000 eggs in a day [7]. These eggs passed in human feces can stay viable in soil up to 10 years and need 10 to 15 days time in soil to molt twice before they become infective [1]. After ingesting these eggs, larvae emerge which then undergo extraintestinal migration. These larvae pass through intestinal walls into portal venous system and lymphatics and reach the lungs via hepatic veins and thoracic lymphatic duct, respectively. On reaching the lungs, these larvae make their way into the alveoli from where they ascend the tracheobronchial tree to reach the hypopharynx to be swallowed again [1]. Return of larvae back to intestine completes the extraintestinal migration of larvae and initiates another molting to turn into an adult worm.

**Figure 2:**
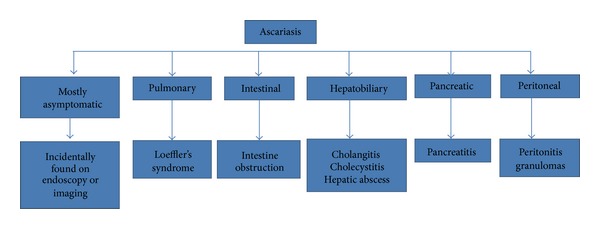
Clinical types of ascariasis.

*A. lumbricoides* infections are largely asymptomatic [1] (see Figure 2) and hence are occasionally identified during routine endoscopic procedures [8, 9] or are seen on radiologic imaging [10]. Symptoms are largely restricted to individuals with high worm load [1].

## 4. Pulmonary Ascariasis

Pulmonary ascariasis manifesting as *A. lumbricoides* pneumonia also known as Loeffler's syndrome occurs after 4 to 16 days of ingesting embryonated *A. lumbricoides* eggs. *A. lumbricoides* pneumonia, a self-limiting illness, is a result of hypersensitivity reaction to *A. lumbricoides* larvae migrating through the lung. Clinical manifestations are cough, dyspnea, fever, and occasionally hemoptysis in individuals with heavy worm loads [1]. Skin urticarial rash may also accompany these symptoms. Chest X-ray reveals rounded infiltrates with peripheral eosinophilia.

## 5. Intestinal Ascariasis

Intestinal ascariasis is generally asymptomatic in individuals with low worm load or may be incidentally discovered on endoscopic procedures as in our case. It can cause vague abdominal symptoms like abdominal pain, distension, nausea, and diarrhea [1]. Careful history can reveal the presence of *A. lumbricoides* worms in stool and vomitus or at times of the passage of these worms even through nostrils. In endemic areas, individuals with heavy worm load can frequently present as intestinal obstruction [11]. A review of a total of 311 cases over a span of 25 years between 1963 and 1988 by Ochoa [11] revealed that heavy intestinal *A. lumbricoides* infestation coupled with ability of *A. lumbricoides* worms to interlace and form huge masses can frequently lead to development of classical symptoms of intestinal obstruction like abdominal pain with vomiting and constipation. Intestinal obstruction also can be complicated by the development of volvulus, volvulus with gangrene and perforation, intussusception, and appendicitis. Intestinal obstruction is managed conservatively with nasogastric suction, anthelmintic treatment, and fluid-electrolyte replacement or in more serious cases with varied surgical procedures like extraluminal manual advancement, enterotomy, bowel resection, and appendectomy [11].

## 6. Hepatobiliary and Pancreatic Ascariasis

Hepatobiliary and pancreatic ascariasis results from entry of *A. lumbricoides* into ampullary orifice in duodenum. Due to small size of ducts, it is less common in children. *A. lumbricoides* worms can ascend into common bile duct, pancreatic duct, or even intrahepatic ducts, and depending on the location, symptoms vary over a wide clinical spectrum of biliary colic, acalculous cholecystitis, acute cholangitis, acute pancreatitis, and hepatic abscess [1]. Ultrasonography and endoscopic retrograde cholangiopancreatography (ERCP) often serve as diagnostic procedures with an additional therapeutic advantage with ERCP [12, 13].

## 7. Peritoneal Ascariasis

Peritoneal ascariasis is a less common complication of ascariasis, which can present as acute peritonitis or chronic peritoneal granulomas resembling tubercular granulomas. 

While adult *A. lumbricoides* worms are often identified by their characteristic appearance, *A. lumbricoides* eggs are easily identified by direct smear examination of a stool sample from an infected individual. Depending on the symptomatology plain radiographs, ultrasonograms and endoscopic procedures including ERCP can be useful.

Treatment of ascariasis includes single dose of Mebendazole or Albendazole or Pyrantel pamoate. A systematic review and meta-analysis of anthelmintic drugs by Keiser J. and Utzinger J. demonstrated high cure rates of single doses of the abovementioned drugs against *A. lumbricoides* [14]. WHO has also emphasized on adapting preventive chemotherapy strategy for the control of ascariasis, trichuriasis, and hookworm infection in high endemic countries. Previous concerns of anthelmintic treatment safety in pregnancy now have been clarified by the work of Torlesse and Hodges [15, 16] and a prospective case control study by de Silva et al. [17]. Anthelmintic treatments are safe during pregnancy provided that treatment is started after the first trimester of pregnancy [18].
